# Supplementary material for: Distinct autoreactive CD19– plasma cell subsets accumulate in lupus-prone mice
Source: Nat Commun. 2025 Nov 10;16:9901. doi: 10.1038/s41467-025-65906-6 (PMC12603280; doi:10.1038/s41467-025-65906-6)
Supplement: Supplementary file 1 — Supplementary Information [file 41467_2025_65906_MOESM1_ESM.pdf]

SUPPLEMENTARY INFORMATION

Distinct Autoreactive CD19^{-} Plasma Cell Subsets Accumulate in Lupus-prone Mice

Van Duc Dang[1,2,3,†], Franziska Szelinski[1,2,†], Elodie Mohr[1], Tuan Anh Le[1,2], Jacob Ritter[1,2], Annika Wiedemann[1,2], Marta Ferreira-Gomes[1], Gabriela Maria Guerra[1], Pawel Durek[1], Frederik Heinrich[1], Hector Rincon-Arevalo[1,2,4,5], Ana-Luisa Stefanski[1,2], Eva Schrezenmeier[1,4,6], Van T. Hoang[7], Hong-Nhung Dao[7], Soeren Ocvirk[8], Qingyu Cheng[1,2], Falk Hiepe[1,2], Christian Hipfl[9], Sebastian Hardt[9], Max Löhning[1,2], Liem Thanh Nguyen[7,10], Mir-Farzin Mashreghi[1,11], Simon Fillatreau[12,13,14], Thomas Dörner[1,2] and Andreia C. Lino[1,2,*]

[1] Deutsches Rheuma-Forschungszentrum, a Leibniz Institute, Charitéplatz 1, 10117 Berlin, Germany

[2] Department of Rheumatology and Clinical Immunology, Charité Universitätsmedizin Berlin, corporate member of Freie Universität Berlin and Humboldt-Universität zu Berlin, Berlin, Germany

[3] Faculty of Biology, VNU University of Science, Vietnam National University, Hanoi, Vietnam

[4] Department of Medicine/Nephrology and Medical Intensive Care, Charité-Universitätsmedizin Berlin, corporate member of Freie Universität Berlin and Humboldt-Universität zu Berlin, Berlin, Germany

[5] Grupo de Inmunología Celular e Inmunogenética, Facultad de Medicina, Instituto de Investigaciones Médicas, Universidad de Antioquia UdeA, Medellín, Colombia

[6] Berlin Institute of Health at Charité – Universitätsmedizin Berlin

[7] Vinmec Research Institute of Stem Cell and Gene Technology, College of Health Sciences, VinUniversity, Vinhomes Ocean Park, Hanoi, Vietnam

[8] Intestinal Microbiology Research Group, Department of Molecular Toxicology, German Institute of Human Nutrition Potsdam-Rehbruecke, Nuthetal, Germany

[9] Centre for Musculoskeletal Surgery, Department of Orthopedics, Charité Universitätsmedizin Berlin, Berlin, Germany

[10] Vinmec Health Care System, Hanoi, Vietnam

[11] German Center for Child and Adolescent Health (DZKJ), partner site Berlin, Berlin, Germany

[12] Institut Necker Enfants Malades, INSERM U1151-CNRS UMR 8253, Paris, France

[13] Faculté de Médecine, Université de Paris, Paris, France

[14] AP-HP, Hôpital Necker Enfants Malades, Paris, France

† These authors contributed equally to this work

* Correspondence: Andreia C. Lino

Deutsches Rheuma-Forschungszentrum, a Leibniz Institute

Charitéplatz 1, 10117 Berlin, Germany

E-mail: andreia.lino@drfz.de or andreialino79@gmail.com

Additional information

Additional information includes:

- Eight figures. **Supplementary Fig. 1 – 8**
- Six tables: **Supplementary tables 1 – 6**

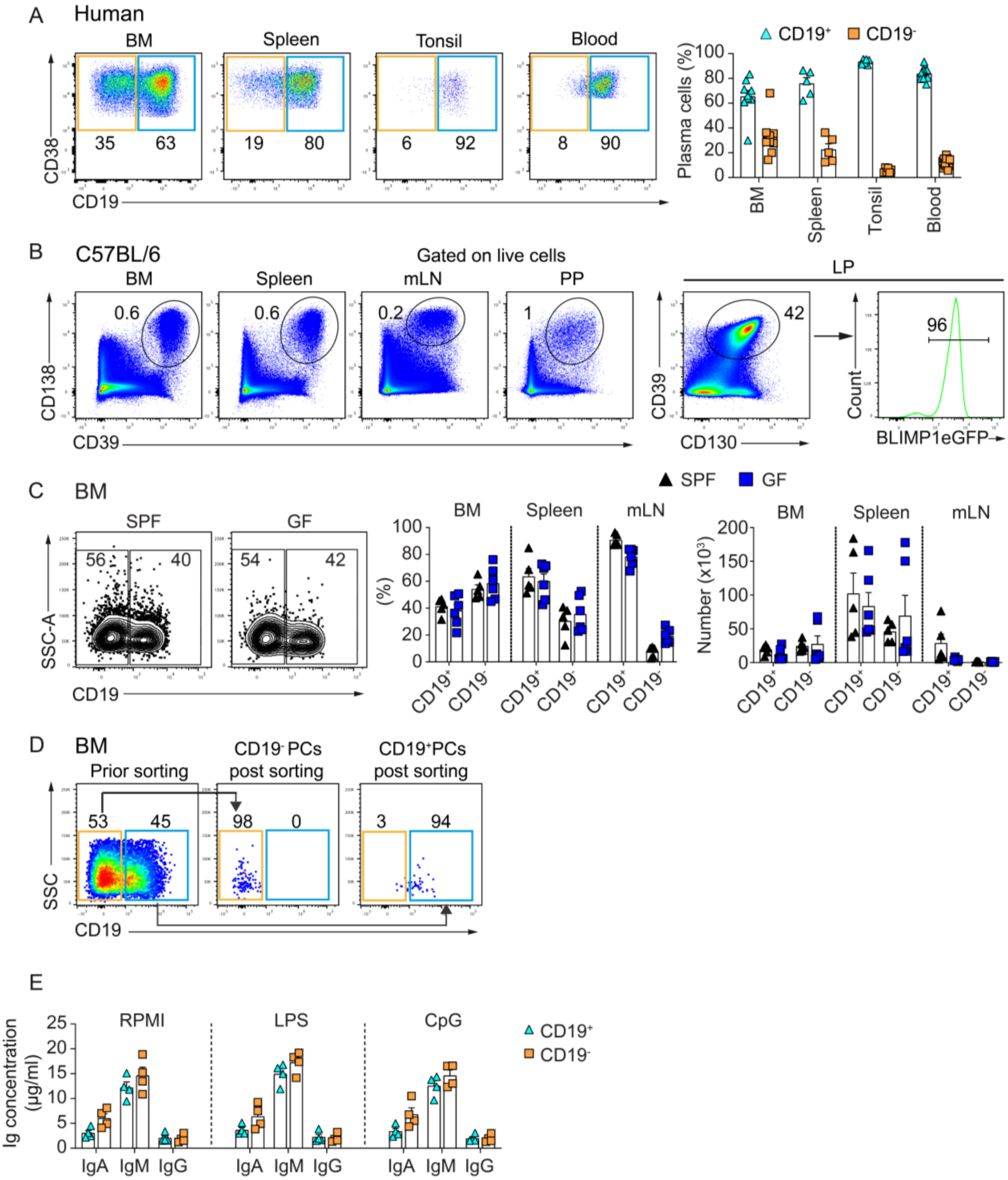

Supplementary Fig. 1. CD19⁻ PCs are enriched in the BM of human and mouse and accumulate with age in mouse

(A) Representative FACS plots (left) show the lack of CD19 expression identifies a PC subpopulation in human bone marrow (BM), spleen, tonsil, and peripheral blood. Cells were gated on total PCs ($CD27^{++}CD38^{++}CD3^{-}CD14^{-}$ live cells)[1, 2]. The graph (right) shows the frequency of $CD19^{+}$ and $CD19^{-}$ PCs in indicated organs. Data show compilation of nine, five, six, and 14 donors for BM, spleen, tonsil, and peripheral blood, respectively.

(B) Representative FACS plots show the identification of total PCs ($CD39^+CD138^+$) gated on live cells (Lymphocytes/Single cells/Propidium iodide negative) in indicated organs. LP PCs were gated as $CD39^+CD130^+$ since CD138 detection was impaired due to enzymatic digestion with collagenase[1].

(C) Representative FACS plots (left) show the frequencies of $CD19^+$ and $CD19^–$ PCs in BM from specific pathogen free (SPF) and germ free (GF) mice. Graphs (right) show the frequency and absolute number of $CD19^+$ and $CD19^–$ PCs in BM, spleen, and mLN from SPF compared with GF mice. Cells were gated on total PCs ($CD39^+CD138^+$) as indicated in Fig. 1A and Supplementary Fig. 1B.

(D) Representative FACS plots show $CD19^+$ and $CD19^–$ BMPCs before and after FACS sorting for ELISPOT assays. Prior sorting plot was gated on BIN^- ($DAPI^-CD3^-CD11b^-CD11c^-$) $CD39^+CD138^+$.

(E) The graph shows the concentration of IgA, IgM and IgG in the supernatants at 24 hours after cultured in RPMI, LPS or CpG of sorted BM $CD19^+$ or $CD19^–$ PCs as indicated in Supplementary Fig. 1D. IgA, IgM and IgG concentrations were determined by ELISAs.

Experiments were performed with naive C57BL/6 mice (B-E). Data show compilation of three (B), two (C), five (D) and four (E) independent experiments; n=11 (BM, spleen, and mLN) and 5 mice (PP and LP) (B), n=5 (SPF) and 6 (GF) mice (C), n=15 mice (D), and n=73 mice (E). Data show mean ± SEM. Bone marrow (BM), mesenteric lymph node (mLN), Peyer's patches (PP), and lamina propria (LP).

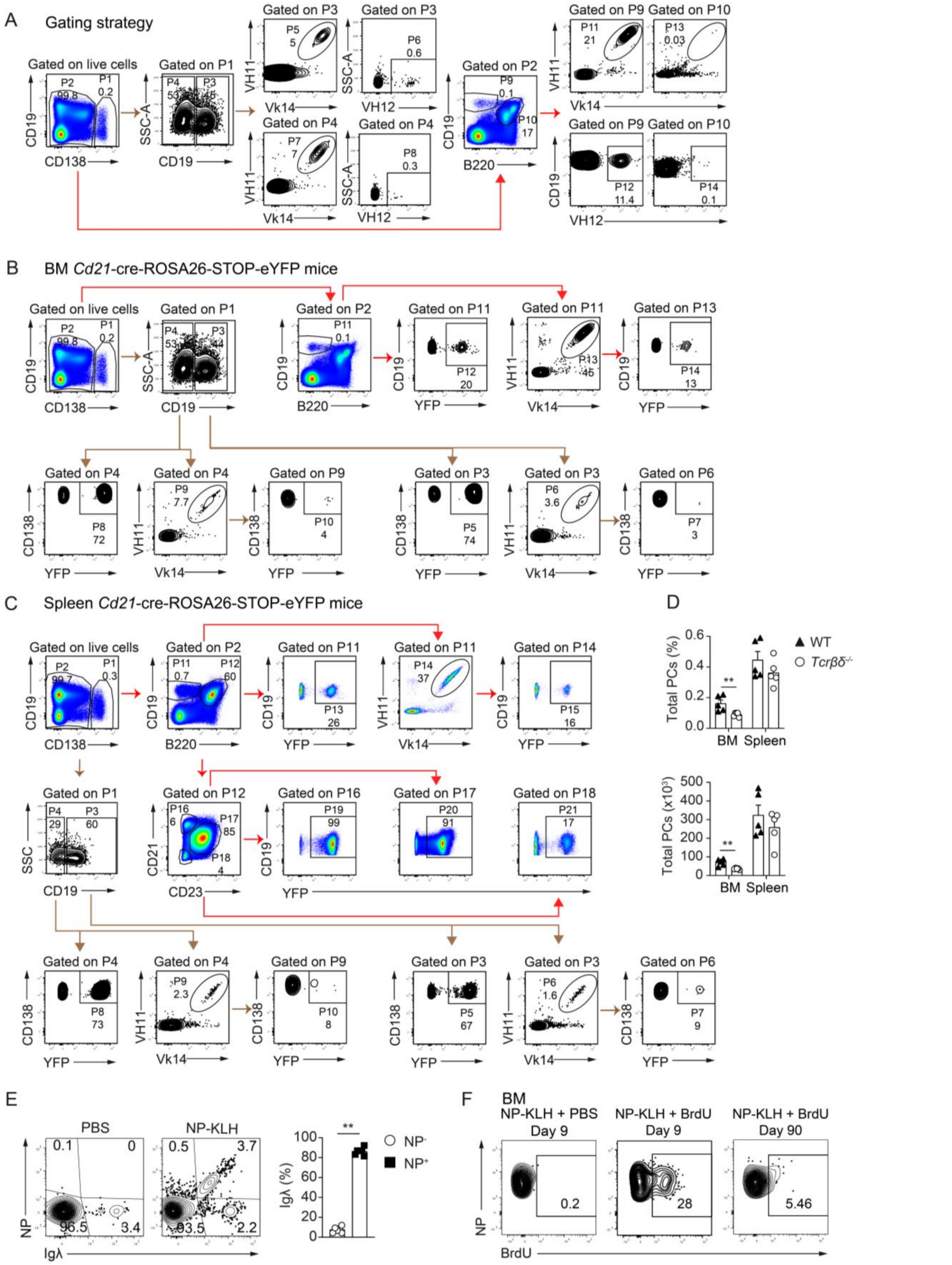

Supplementary Fig. 2. CD19⁻ BMPCs derive from various B cell subsets upon T-dependent and -independent reactions

(A) Gating strategy identifying CD19^{+} (P3) and CD19$^{–}$ BMPCs (P4), B-1 (P9) and B-2 (P10) B cells for the expression of VH11/Vk14 and VH12 in BM of naive C57BL/6 mice.

(B-C) Gating strategy identifying YFP^{+} cells in B and PC subsets from BM (B) and spleen (C) of *Cd21*-cre-ROSA26-STOP-eYFP mice. (B) P3, P4 and P11 are CD19^{+} PCs, CD19$^{–}$ PCs, and B-1 cells, respectively. (C) P3, P4, P11, P16, P17, and P18 are CD19^{+} PCs, CD19$^{–}$ PCs, B-1, marginal zone, follicular and double negative B cell subsets, respectively.

(D) Graphs showing the frequency (top) and absolute number (bottom) of total PCs (CD39^{+}CD138^{+}) in BM and spleen from T cell deficient mice (*Tcrβδ*$^{-/-}$) compared with WT C57BL/6 controls.

(E) Representative FACS plots (left) show NP-specific and Igλ expression by BMPCs (CD39^{+}CD138^{+}) at days 90 after NP-KLH immunization from C57BL/6 mice. The graph (right) shows the frequency of Igλ^{+} PCs in NP^{+} and NP^{-} BMPCs.

(F) Representative FACS plots show the detection of BrdU^{+} cells in BMPCs in immunized C57BL/6 mice corresponding to Fig. 2G.

Data show compilation of two independent experiments (D-E) (n=5/group for D and n=6 mice for E). Groups were compared using two-tailed non-parametric Mann-Whitney (**p = 0.0079 for D and p = 0.0022 for E) (D-E). P values ≥ 0.05 are not shown. Data show mean ± SEM.

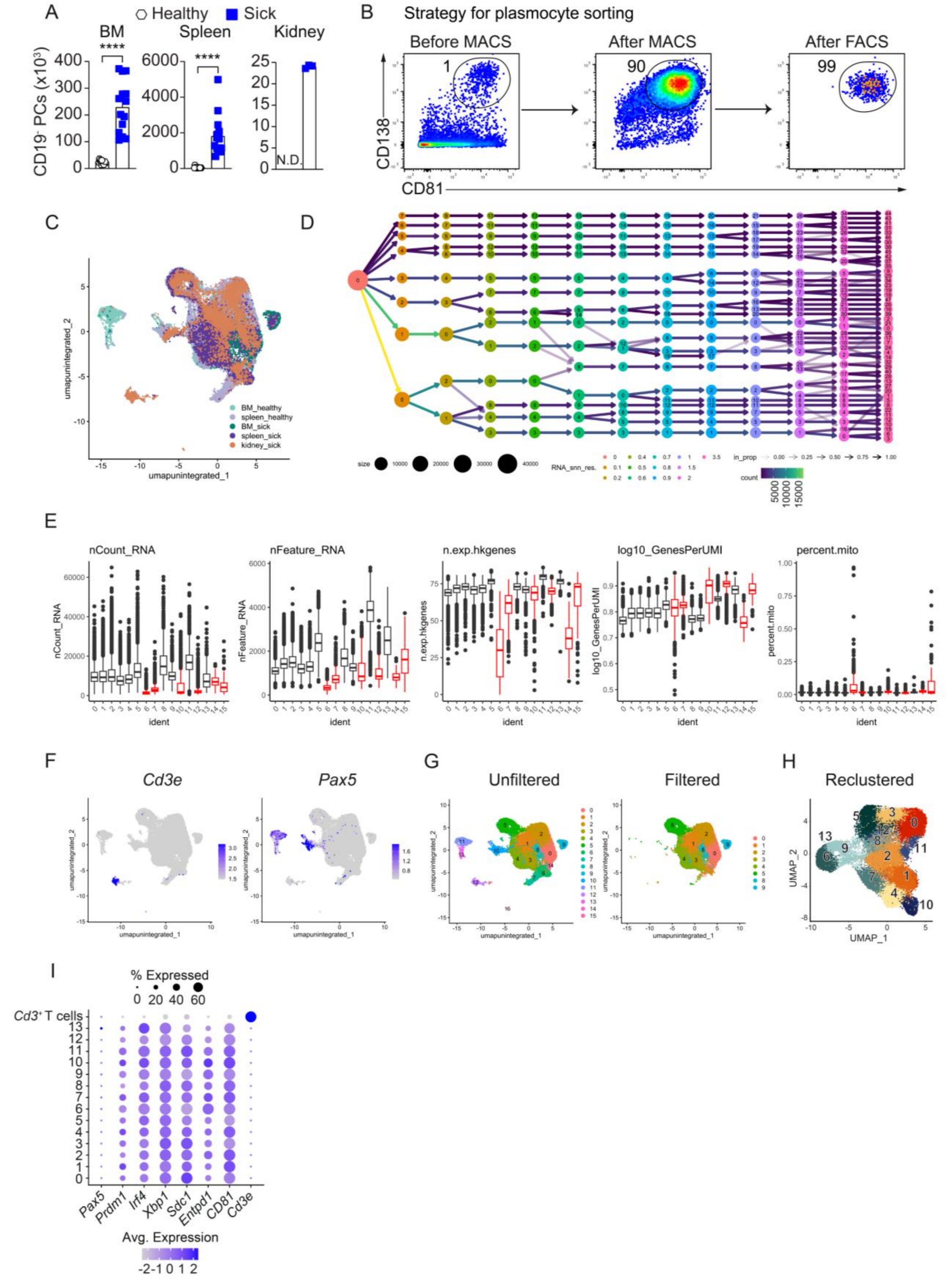

Supplementary Fig. 3. CD19⁻ PCs accumulate in human SLE blood and in target organs of SLE mice

(A) The graphs show number of CD19⁻ PCs in indicated organs of sick compared with healthy *Sle123* mice. CD19⁻ PCs were gated on total PCs (CD39⁺CD138⁺)[1]. Data show four independent

experiments (n=12 healthy mice and n=14 sick mice), except kidney (one experiment, n=3 mice/group). Groups were compared using two-tailed non-parametric Mann-Whitney test (**** $p < 0.0001$).

(B) Strategy for PC sorting. FACS plots show the PC population at the time before MACS, after $CD138^+$ enrichment by MACS, and after FACS sorting.

(C) UMAP of combined data from BM, spleen, and kidney.

(D) The cluster tree shows the relationships between cluster numbers and multiple resolutions.

(E) Boxplots of parameters for quality control

(F) UMAP plots showing the expression of *Cd3e* (left) and *Pax5* (right) across the integrated dataset. The color intensity represents the level of gene expression, with darker shades indicating higher expression levels. These plots highlight the distribution of T cells (*Cd3e*) and non-PC B cells (*Pax5*) in the dataset.

(G) UMAP plots display the clustering of cells before (Unfiltered, left) and after (Filtered, right) quality control filtering. Each color represents a different cluster, with cluster numbers indicated on the plots.

(H) UMAP plot of the re-clustered dataset after filtering, with each color representing a different cluster. This plot demonstrates the refined clustering of immune cell populations following data cleaning and re-clustering.

(I) Bubble plot illustrating the expression levels of key PC markers across the identified clusters in the re-clustered filtered dataset. Excluded $Cd3e^+$ positive T cells were included as non-PC reference in this plot solely for the purpose of comparison. The x-axis represents the genes (*Pax5, Prdm1*, *Irf4*, *Xbp1*, *Sdc1*, *Entpd1*, *Cd81* and *Cd3e*), and the y-axis represents the PC clusters (0-13) and $Cd3e^+$ cells. The size of the dots indicates the percentage of cells expressing each gene within the cluster, and the color intensity represents the average expression level.

(B-I) Experiments and analyses were performed with *Sle123* mice. Data show compilation of n=6 mice/group (BM and Spleen) and n=13 mice (Kidney).

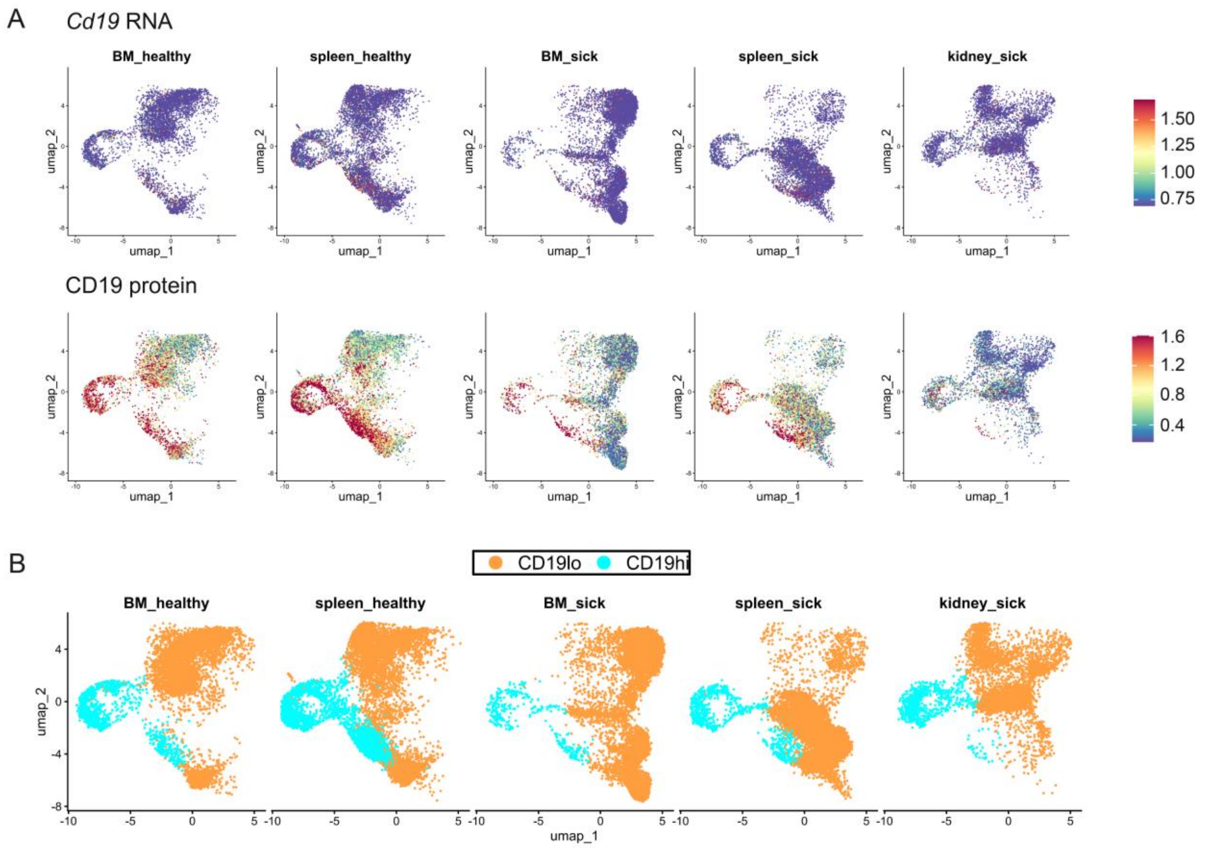

Supplementary Fig. 4. Lupus-associated PC subsets show reduced CD19 expression and distinct regulatory profiles

(A) UMAP plots show the expression of *Cd19* mRNA (top) and CD19 protein (bottom) by indicated clusters and organs from healthy and sick *Sle123* mice. Color scale shows the average expression.

(B) UMAP plots show the $CD19^{lo}$ and $CD19^{hi}$ regions across tissues from healthy and sick *Sle123* mice.

Data show compilation of n=6 mice/group (BM and Spleen) and n=13 mice (Kidney).

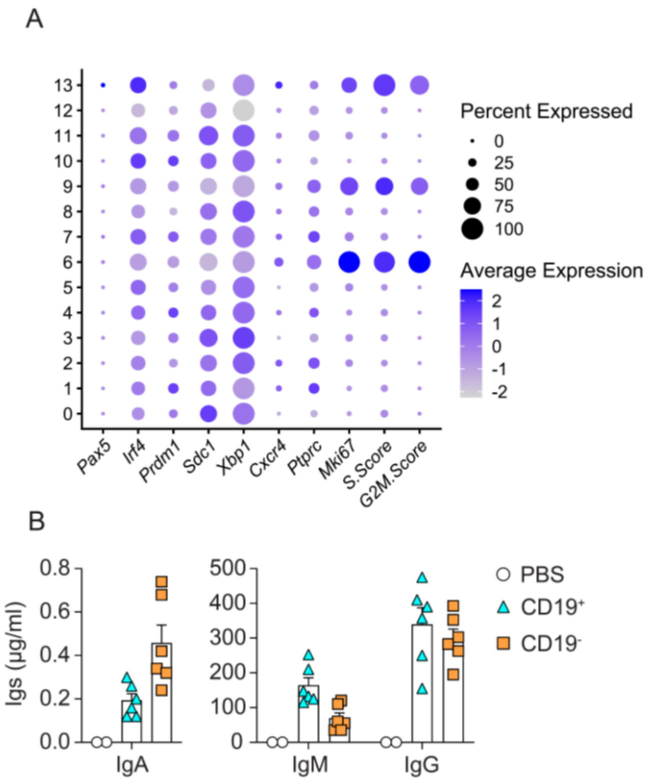

Supplementary Fig. 5. CD19⁻ PCs are derived from CD19+ PCs

(A) Bubble plot shows the expression of indicated genes associated with PCs and cell cycle scores across clusters. Color scale shows the average expression. Bubble sizes show the frequency of cells expressing indicated genes by indicated clusters. Experiments and analyses were performed with *Sle123* mice. Data show compilation of n=6 mice/group (BM and Spleen) and n=13 mice (Kidney).

(B) The graph shows the concentration of IgA, IgM and IgG from sera of *Rag*$^{-/-}$ mice received PBS, sorted CD19^{+} or CD19^{-} PCs at day 7 after adoptive transfer as indicated in Fig. 5B. IgA, IgM and IgG concentrations were determined by ELISAs. Data show compilation of two independent experiments (n=6 mice/group). Data show mean ± SEM.

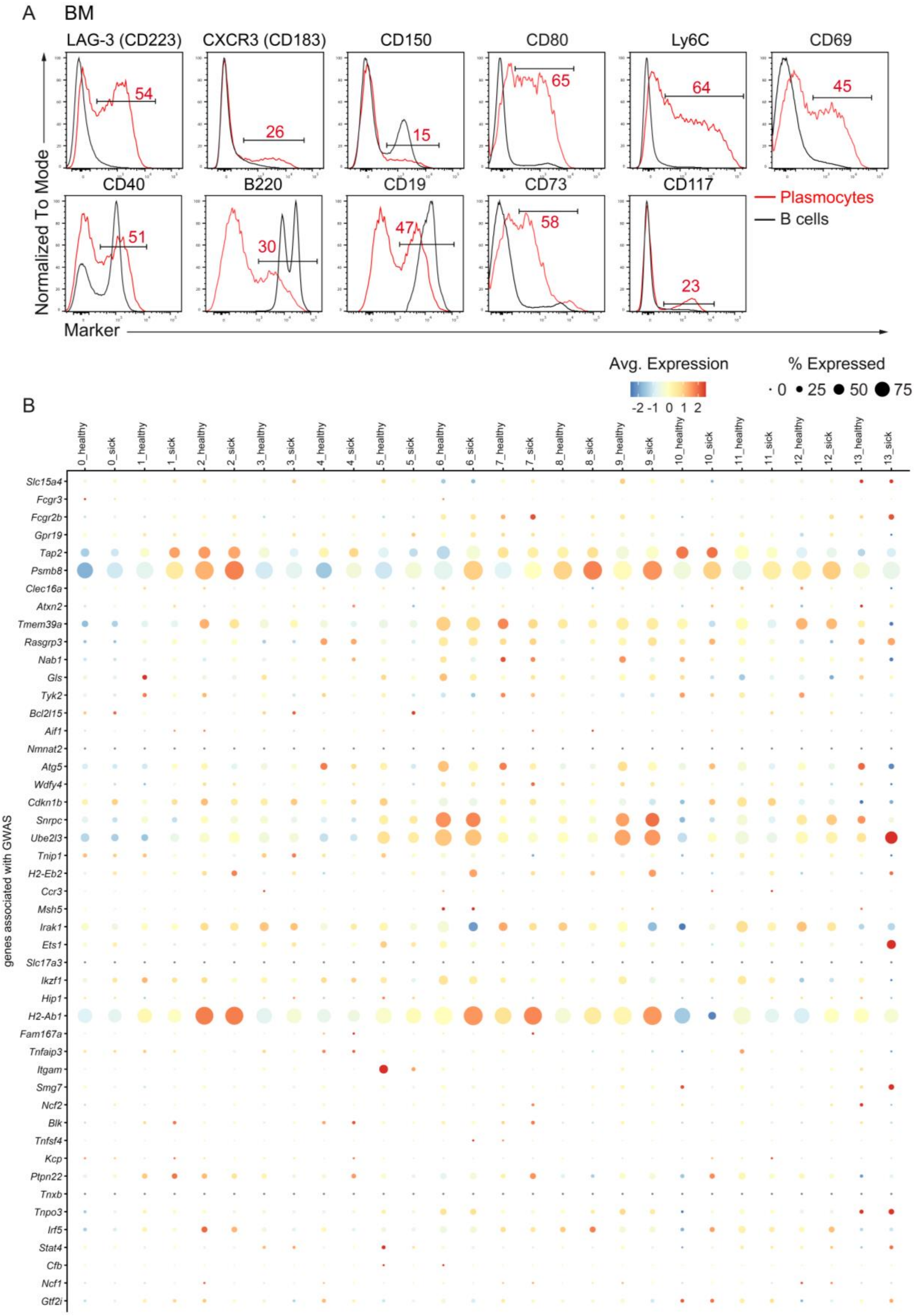

Supplementary Fig. 6. Single cell transcriptomic profiling reveals disease- and organ-specific PCs in SLE mice

(A) Histograms showing indicated surface molecule expression by BM CD138^{+}BLIMP-1eGFP^{+} PCs (red) and CD19^{+}CD138^{-}BLIMP-1eGFP^{-} B cells (black) obtained from the LEGEND

screening experiment of 255 surface proteins. Experiments were performed with *prdm1*eGFP mice.

(B) Bubble plot shows the expression of the full list of top 50 mouse orthologs of human genes associated with systemic lupus erythematosus (GWAS, MONDO:0007915), ranked by minimal SNP P-value. Bubble size indicates the percentage of cells in that sample with nonzero expression of the given gene, and bubble color encodes the average scaled expression level. Experiments and analyses were performed with *Sle123* mice. Data show compilation of n=6 mice/group (BM and Spleen) and n=13 mice (Kidney).

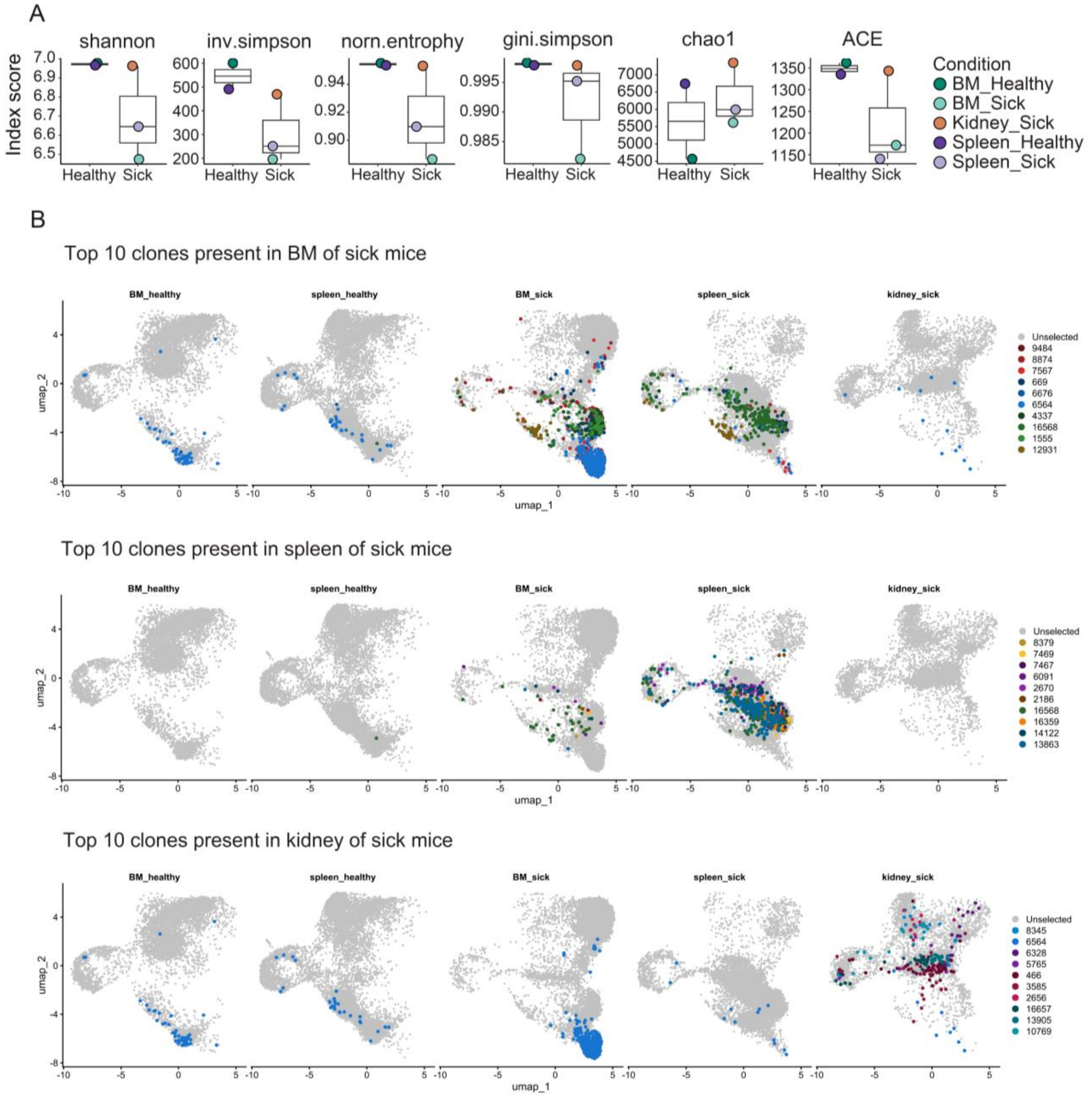

Supplementary Fig. 7. The most expanded BCR clones in kidney of lupus mice are highly mutated

(A) Box plots illustrate the differences in BCR diversity between healthy and sick mice, as measured by various diversity indices include Shannon, Inverse Simpson, Normalized Entropy, Gini-Simpson, Chao1, and ACE across BM, kidney, and spleen tissues. Each box plot represents the distribution of index scores with the median indicated by the central line, the interquartile range by the box, and the overall range by the whiskers.

(B) UMAP plots showing the distribution of the top 10 clones in BM, spleen, and kidney across different conditions (BM_healthy, spleen_healthy, BM_sick, spleen_sick, and kidney_sick). Each plot highlights the clonal expansion and distribution for each specific tissue and condition.

Experiments and analyses were performed with *Sle123* mice. Data show compilation of n=6 mice/group (BM and Spleen) and n=13 mice (Kidney).

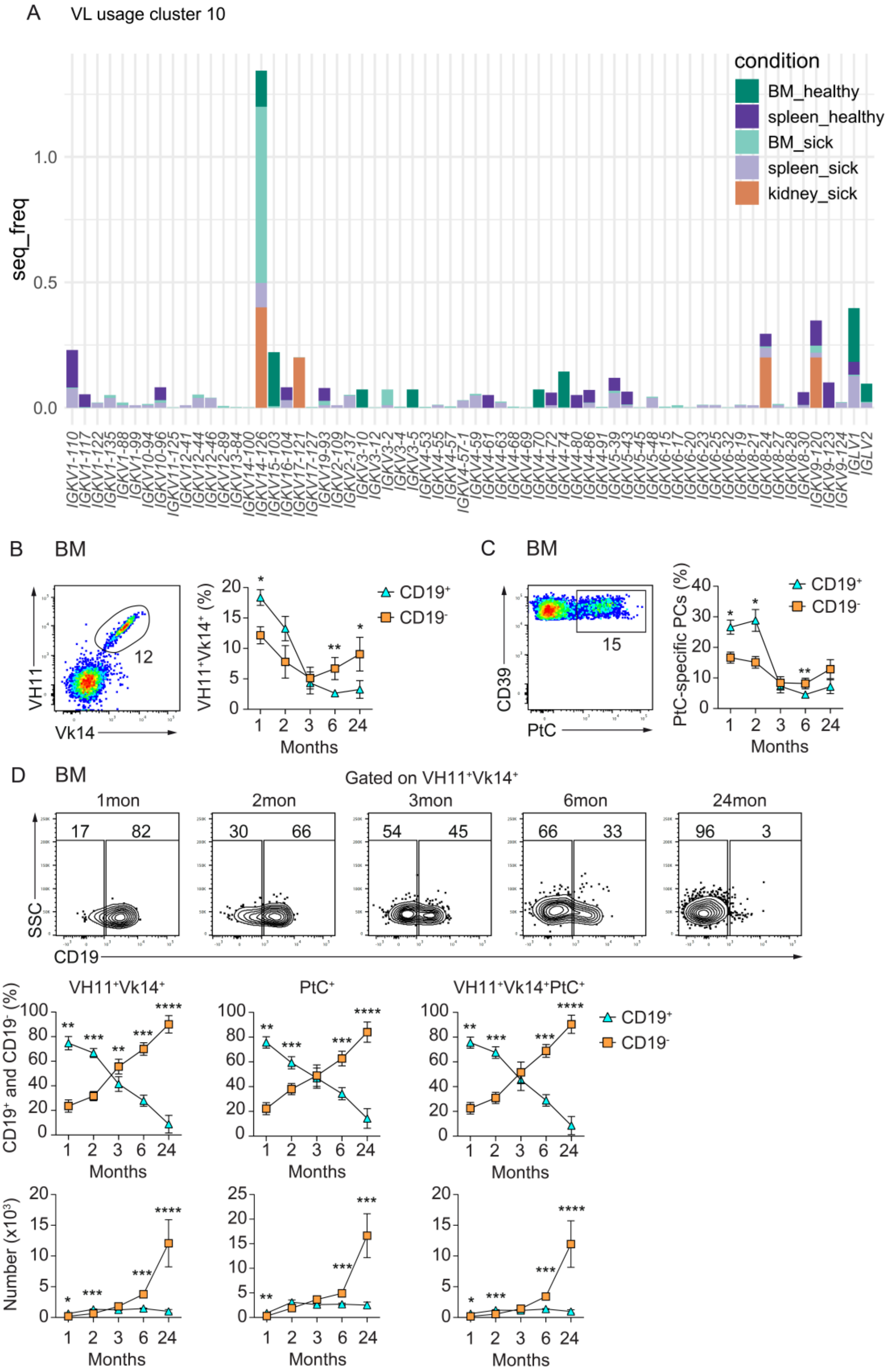

Supplementary Fig. 8. Phosphatidylcholine-specific CD19⁻ B-1 derived PCs accumulate in BM of sick SLE mice

(A) Stacked bar plot illustrating the Vk light chain gene usage across tissues and conditions. Each bar represents the frequency of specific Vk genes utilized in each condition, highlighting

variations in gene usage patterns. Experiments were performed with *Sle123* mice. Experiments and analyses were performed with *Sle123* mice. Data show compilation of n=6 mice (BM and Spleen) and n=13 mice (Kidney).

(B) Representative FACS plot (left) and graph (right) show the presence of $VH11^{+}Vk14^{+}$ cells in $CD19^{+}$ and $CD19^{-}$ PCs in BM. $CD19^{+}$ and $CD19^{-}$ BMPCs were gated on total PCs ($CD39^{+}CD138^{+}$) as indicated in Fig. 1A.

(C) Representative FACS plot (left) and graph (right) show the frequency of Phosphatidylcholine (PtC)-specific PCs in $CD19^{+}$ and $CD19^{-}$ PCs in BM. $CD19^{+}$ and $CD19^{-}$ BMPCs were gated on total PCs ($CD39^{+}CD138^{+}$) as indicated in Fig. 1A.

(D) Representative FACS plots (top) show the frequency of $CD19^{+}$ and $CD19^{-}$ PCs in $VH11^{+}Vk14^{+}$ PCs ($CD39^{+}CD138^{+}VH11^{+}Vk14^{+}$), graphs (middle) show the frequency of $CD19^{+}$ and $CD19^{-}$ in $VH11^{+}Vk14^{+}$ ($CD39^{+}CD138^{+}VH11^{+}Vk14^{+}$) (left), PtC-specific ($CD39^{+}CD138^{+}PtC^{+}$) (middle) and $VH11^{+}Vk14^{+}$ PtC-specific ($CD39^{+}CD138^{+}VH11^{+}Vk14^{+}PtC^{+}$) (right) PCs, and graphs (bottom) show the absolute number of $CD19^{+}VH11^{+}Vk14^{+}$, $CD19^{-}VH11^{+}Vk14^{+}$, $CD19^{+}PtC^{+}$, $CD19^{-}PtC^{+}$, $CD19^{+}VH11^{+}Vk14^{+}$ PtC^{+}, and $CD19^{-}$ $VH11^{+}Vk14^{+}$ PtC^{+} in BM.

(B-D) Experiments were performed with naive C57BL/6 mice. Data show compilation of three independent experiments; n=5, 7, 7, 8 and 9 mice/group for 1, 2, 3, 6, and 24 months old, respectively. Groups were compared using two tailed non-parametric Mann-Whitney test (*p < 0.05, **p < 0.01, *** p < 0.001, **** p < 0.0001). P values ≥ 0.05 are not shown. Data show mean ± SEM.

Supplementary Table 1: Characteristics of healthy donors and patients with systemic lupus erythematosus (SLE)

IDs	Gender	Disease	cSLEDAI	SLEDAI	
Healthy Donors average age 35.88					
1	f	-			
2	f	-			
3	m	-			
4	f	-			
5	m	-			
6	m	-			
7	f	-			
8	f	-			
SLE patients average age 33.70			cSLEDAI	SLEDAI	
1	f	SLE	0	4	mild
2	f	SLE	2	6	active
3	f	SLE	2	6	active
4	f	SLE	0	2	mild
5	f	SLE	8	12	active
6	f	SLE	0	0	quiescent
7	f	SLE	0	4	mild
8	f	SLE	3	7	active
9	f	SLE	6	10	active
10	f	SLE	-	-	

SLEDAI, Systemic Lupus Erythematosus Disease Activity Index; cSLEDAI, clinical SLEDAI. Disease activity categories: active, SLEDAI ≥ 6; mild, SLEDAI 1–5; quiescent, SLEDAI 0.

Supplementary Table 2: Mouse Specific Monoclonal Antibodies: List including clone, conjugate and source information

Target	Conjugat	Clone	Source	Catalog no	Conc.
B220/CD45R	BV711	RA3-6B2	BioLegend	103255	1:400
B220/CD45R	PE	RA3-6B2	BioLegend	103208	1:400
CD2	PE	RM2-5	BioLegend	100107	1:200
CD2	APC-Vio770	REA959/RM2-5	Miltenyi Biotec	130-115-962	1:200
CD3	APC/Cy7	17A2	BioLegend	100221	1:100
CD3	BV421	145-2C11	BioLegend	100341	1:200
CD5	FITC	53-7.3	BD Biosciences	553021	1:100
CD5	BV510	53-7.3	BioLegend	100627	1:100
CD11b	Pacific Blue	M1/70	BioLegend	101224	1:400
CD11b	APC/Cy7	M1/70	BioLegend	101226	1:200

CD11c	Pacific Blue	N418	BioLegend	117322	1:100
CD16/CD32 (Fc Block)	-	Clone 2.4G2	DRFZ	Non-commercial	30µg/mL
CD19	BV785	6D5	BioLegend	115543	1:200
CD19	BUV737	1D3	BD Biosciences	612781	1:200
CD20	PE	SA275A11	BioLegend	150409	1:200
CD21/35	FITC	7G6	BD Biosciences	553818	1:200
CD21/35	PE/Cy7	7E9	BioLegend	123420	1:200
CD23	Biotin	B3B4	BD Biosciences	553137	1:400
CD23	Alexa 647	B3B4	BioLegend	101612	1:400
CD38	PE	90	BioLegend	102707	1:200
CD39	PE/Dazzle 594	Duha59	BioLegend	143811	1:400
CD39	APC	Duha59	BioLegend	143810	1:400
CD40	PE	3/23	BioLegend	124609	1:200
CD49b	PE	HMα2	BioLegend	103506	1:400
CD69	PE	H1.2F3	BioLegend	104507	1:200
CD69	FITC	REA937/H1.2F3	Miltenyi Biotec	130-115-574	1:100
CD73	PE	TY/11.8	BioLegend	127205	1:200
CD73	APC-Vio770	REA778/TY/11.8	Miltenyi Biotec	130-111-520	1:100
CD80	BV650	16-10A1	BioLegend	104731	1:100
CD80	PE	16-10A1	BioLegend	104707	1:200
CD81	PE/Cy7	Eat-2	BioLegend	104913	1:200
CD86	BV785	GL-1	BioLegend	105043	1:200
CD86	PE	PO3	BioLegend	105105	1:200
CD117	PE	2B8	BioLegend	105807	1:200
CD130	PE	4H1B35	BioLegend	149403	1:100
CD138	PE	281-2	BD Biosciences	553714	1:400
CD138	BV421	281-2	BioLegend	142523	1:800
CD150	PE	TC15-12F12.2	BioLegend	115903	1:200
CD326	BV510	G8.8	BioLegend	118231	1:200
CD326	APC/Cy7	G8.8	BioLegend	118218	1:200
CD352	VioBright B515	REA1097/13G3	Miltenyi Biotec	130-118-731	1:150
CD352	PE	330-AJ	BioLegend	134605	1:200
CD365	PE	RMT1-4	BioLegend	119505	1:100
CXCR3	APC	CXCR3-173	BioLegend	126512	1:200
Igλ	Alexa 647	Clone LS136	DRFZ	Non-commercial	1:200
JAML	Alexa 647	4E10	BioLegend	128506	1:100
Ki-67	APC	16A8	BioLegend	652405	1:200
LAG-3 (CD223)	PE/Cy7	eBioC9B7W	Thermo Fisher	25-2231-82	1:150
Ly6C	PE	HK1.4	BioLegend	128007	1:400
MHC-II	FITC	Clone M5/114	DRFZ	Non-commercial	1:200
TIGIT	PE	1G9	BioLegend	142103	1:200
LEGENDScreen Mouse PE Kit	PE	-	BioLegend	700005	-

VH11	PE	Clone P18-3H7	Gifted by Kyoko Hayakawa	Non-commercial	1:400
Vk14	Pacific Orange	Clone P18-13B5	Gifted by Kyoko Hayakawa	Non-commercial	1:100
VH12	APC	Clone 5C5	Gifted by Klaus Rajewsky	Non-commercial	1:200

Supplementary Table: 3 DNA-barcoded Antibodies (CITE-seq): List including clone, conjugate and source information

Target	Conjugat	Clone	Source	Catalog no	Conc.
CD19	TotalSeq-C0093	6D5	BioLegend	115571	1:400
CD39	TotalSeq-C0834	Duha59	BioLegend	143815	1:100
CD40	TotalSeq-C0903	3/23	BioLegend	124635	1:400
CD45R/B220	TotalSeq-C0103	RA3-6B2	BioLegend	103273	1:200
CD69	TotalSeq-C0197	H1.2F3	BioLegend	104551	1:200
CD73	TotalSeq-C0077	TY/11.8	BioLegend	127237	1:200
CD80	TotalSeq-C0849	16-10A1	BioLegend	104755	1:200
CD183 (CXCR3)	TotalSeq-C0228	CXCR3-173	BioLegend	126545	1:200
CD223 (LAG-3)	TotalSeq-C0378	C9B7W	BioLegend	125237	1:200
CD326	TotalSeq-C0449	G8.8	BioLegend	118243	1:200
CD117 (c-Kit)	TotalSeq-C0012	2B8	BioLegend	105851	1:400
CD150 (SLAM)	TotalSeq-C0203	TC15-12F12.2	BioLegend	115947	1:400
Ly6C	TotalSeq-C0013	HK1.4	BioLegend	128051	1:400

Supplementary Table 4: Human Specific Monoclonal Antibodies: List including clone, conjugate and source information

Target	Conjugat	Clone	Source	Catalog no	Conc.
CD3	BUV395	UCHT1	BD Biosciences	563546	1:50
CD14	BUV395	M5E2	BD Biosciences	740286	1:50
CD19	BV711	SJ25C1	BD Biosciences	563038	1:20
CD20	BV510	2H7	BioLegend	302340	1:20
CD27	BV786	L128	BD Biosciences	563327	1:25
CD38	APC/Cy7	HIT2	BioLegend	303534	1:500
CD39	BV421	A1	BioLegend	328214	1:20
CD81	FITC	5A6	BioLegend	349504	1:20
CD130	PE/Cy7	2E1B02	BioLegend	362007	1:20
CD138	BUV737	MI15	BD Biosciences	564393	1:20
CD326	BV605	9C4	BioLegend	324224	1:20

FcR Blocking Reagent	-	-	Miltenyi Biotec	130-059-901	1:20

Supplementary Table 5: Additional Antibodies: List including clone, conjugate and source information

Goat Anti-Mouse Ig (H+L)	-	polyclonal	SouthernBiotech	1010-01	1:1000
Anti-Mouse IgM-AP	AP	polyclonal	SouthernBiotech	1020-04	1:1000
Anti-Mouse IgA-AP	AP	polyclonal	SouthernBiotech	1040-04	1:1000
Anti-Mouse IgG-AP	AP	polyclonal	SouthernBiotech	1030-04	1:1000
Anti-PE MicroBeads	Beads	-	Miltenyi Biotec	130-048-801	1:10
Anti-PE MicroBeads UltraPure	Beads	-	Miltenyi Biotec	130-105-639	1:10
Streptavidin-BV650	BV650	-	BioLegend	405232	1:400
Streptavidin-Alexa 488	Alexa 488	-	Thermo Fisher	S32354	1:400

Supplementary Table 6: List of Software and Packages

**Tool / Package**	**Version**	**Source**
FlowJo	10	BD Biosciences
GraphPad Prism	9	GraphPad Software
Softmax Pro		Molecular Devices
CellRanger	5.0.0	10x Genomics
R	04.03.2001	CRAN
airr	01.05.2000	CRAN
alakazam	01.03.2000	CRAN
AnnotationDbi	1.64.1	Bioconductor
AnnotationFilter	1.26.0	Bioconductor
ArchR	1.0.2	GitHub (Greenleaf Lab)
beepr	1.3	CRAN
Biobase	2.60.0	Bioconductor
BiocGenerics	0.46.0	Bioconductor
biomaRt	2.58.2	Bioconductor
Cairo	01.06.2002	CRAN
circlize	0.4.16	CRAN
clusterProfiler	04.10.2001	Bioconductor
clustree	0.5.1	CRAN
ComplexHeatmap	2.18.0	Bioconductor
cowplot	01.01.2003	CRAN
data.table	1.15.4	CRAN

DOSE	3.28.2	Bioconductor
dowser	02.02.2000	CRAN
dplyr	01.01.2004	CRAN
DT	0.33	CRAN
enrichplot	1.22.0	Bioconductor
EnsDb.Hsapiens.v79	2.99.0	Bioconductor
ensembldb	2.26.1	Bioconductor
fgsea	1.28.0	Bioconductor
GenomeInfoDb	1.38.8	Bioconductor
GenomicFeatures	1.54.4	Bioconductor
GenomicRanges	1.54.1	Bioconductor
ggalluvial	0.12.5	CRAN
ggplot2	03.05.2001	CRAN
ggraph	02.02.2001	CRAN
ggtree	03.10.2001	Bioconductor
glmGamPoi	1.14.3	Bioconductor
gridExtra	2.3	CRAN
gt	0.10.1	CRAN
gtable	0.3.5	CRAN
gtools	03.09.2005	CRAN
hdf5r	01.03.2010	CRAN
IRanges	2.34.1	Bioconductor
kableExtra	01.04.2000	CRAN
knitr	1.46	CRAN
lubridate	01.09.2003	CRAN
magrittr	2.0.3	CRAN
Matrix	01.06.2005	CRAN
Matrix.utils	0.9.8	CRAN
MatrixGenerics	1.14.0	Bioconductor
matrixStats	01.03.2000	CRAN
msigdbr	07.05.2001	CRAN
Nebulosa	1.15.0	Bioconductor
org.Hs.eg.db	3.18.0	Bioconductor
org.Mm.eg.db	3.18.0	Bioconductor
pacman	0.5.1	CRAN
paletteer	01.06.2000	CRAN
patchwork	01.02.2000	CRAN
pheatmap	1.0.12	CRAN
plyr	01.08.2009	CRAN
pryr	0.1.6	CRAN
purrr	1.0.2	CRAN
RColorBrewer	01.01.2003	CRAN
Rcpp	1.0.12	CRAN
ReactomePA	1.46.0	Bioconductor
readr	02.01.2005	CRAN

readxl	01.04.2003	CRAN
rhdf5	2.46.1	Bioconductor
rstatix	0.7.2	CRAN
Rstudioapi	0.16.0	CRAN
S4Vectors	0.38.2	Bioconductor
scales	01.03.2000	CRAN
scoper	01.03.2000	CRAN
Seurat	05.01.2000	CRAN
SeuratData	0.2.2	GitHub (satijalab)
SeuratObject	5.0.2	CRAN
shazam	01.02.2000	CRAN
SingleCellExperiment	1.24.0	Bioconductor
sp	02.01.2004	CRAN
stringr	01.05.2001	CRAN
SummarizedExperiment	1.32.0	Bioconductor
tibble	03.02.2001	CRAN
tidyr	01.03.2001	CRAN
tidyverse	2.0.0	CRAN
tigger	01.01.2000	CRAN
viridis	0.6.5	CRAN
viridisLite	0.4.2	CRAN
writexl	01.05.2000	CRAN
Immcantation Workflow	04.05.2000	Immcantation.org

References

1. Dang VD*, et al.* CD39 and CD326 Are Bona Fide Markers of Murine and Human Plasma Cells and Identify a Bone Marrow Specific Plasma Cell Subpopulation in Lupus. *Front Immunol* **13**, 873217 (2022).

2. Cossarizza A*, et al.* Guidelines for the use of flow cytometry and cell sorting in immunological studies (third edition). *Eur J Immunol* **51**, 2708–3145 (2021).
