# Supplementary material for: Distinct autoreactive CD19– plasma cell subsets accumulate in lupus-prone mice
Source: Nat Commun. 2025 Nov 10;16:9901. doi: 10.1038/s41467-025-65906-6 (PMC12603280; doi:10.1038/s41467-025-65906-6)
Supplement: Supplementary file 2 — Reporting Summary [file 41467_2025_65906_MOESM2_ESM.pdf]

nature portfolio

Corresponding author(s): Andreia C. Lino

Last updated by author(s): Sep 29, 2025

Reporting Summary

Nature Portfolio wishes to improve the reproducibility of the work that we publish. This form provides structure for consistency and transparency in reporting. For further information on Nature Portfolio policies, see our Editorial Policies and the Editorial Policy Checklist.

Statistics

For all statistical analyses, confirm that the following items are present in the figure legend, table legend, main text, or Methods section.

n/a	Confirmed	
☐	☒	The exact sample size (*n*) for each experimental group/condition, given as a discrete number and unit of measurement
☐	☒	A statement on whether measurements were taken from distinct samples or whether the same sample was measured repeatedly
☐	☒	The statistical test(s) used AND whether they are one- or two-sided<br>*Only common tests should be described solely by name; describe more complex techniques in the Methods section.*
☒	☐	A description of all covariates tested
☐	☒	A description of any assumptions or corrections, such as tests of normality and adjustment for multiple comparisons
☐	☒	A full description of the statistical parameters including central tendency (e.g. means) or other basic estimates (e.g. regression coefficient) AND variation (e.g. standard deviation) or associated estimates of uncertainty (e.g. confidence intervals)
☐	☒	For null hypothesis testing, the test statistic (e.g. *F*, *t*, *r*) with confidence intervals, effect sizes, degrees of freedom and *P* value noted<br>*Give P values as exact values whenever suitable.*
☒	☐	For Bayesian analysis, information on the choice of priors and Markov chain Monte Carlo settings
☒	☐	For hierarchical and complex designs, identification of the appropriate level for tests and full reporting of outcomes
☒	☐	Estimates of effect sizes (e.g. Cohen's *d*, Pearson's *r*), indicating how they were calculated

Our web collection on statistics for biologists contains articles on many of the points above.

Software and code

Policy information about availability of computer code

Data collection	Flow cytometry: BD FACSymphony and BD LSRFortessa machine (BD FACS Diva Software V8.0)<br>Cell sorter: BD FACS sorter Aria II (BD FACS Diva Software V8.0)<br>RNA sequencing: NextSeq2000 device (Illumina),<br>ELISA: BSpectraMax Plus 384 (Molecular Devices)<br>ELISPOT: AID EliSpot Reader System
Data analysis	Flowjo version10 (BD), GraphPad Prism 9 (Graphpad Software), Softmax Pro software<br><br>For the scRNA analysis the follwoing tools were used:<br>CellRanger (v5.0.0), R (version 4.3.1) https://www.R-project.org, and the following packaged: airr (version 1.5.0) CRAN (R 4.3.1), alakazam (version 1.3.0) CRAN (R 4.3.1), AnnotationDbi (version 1.64.1) Bioconductor, AnnotationFilter (version 1.26.0) Bioconductor, ArchR (version 1.0.2) Github (Greenleaf), beepr (version 1.3) CRAN (R 4.2.1), Biobase (version 2.60.0) Bioconductor, BiocGenerics (version 0.46.0) Bioconductor, biomaRt (version 2.58.2) Bioconductor 3.18, Cairo (version 1.6-2) CRAN (R 4.3.2), circlize (version 0.4.16) CRAN (R 4.3.3), clusterProfiler (version 4.10.1) Bioconductor 3.18, clustree (version 0.5.1) CRAN (R 4.3.2), codetools (version 0.2-20) CRAN (R 4.3.3), ComplexHeatmap (version 2.18.0) Bioconductor, cowplot (version 1.1.3) CRAN (R 4.3.3), data.table (version 1.15.4) CRAN (R 4.3.3), DOSE (version 3.28.2) Bioconductor, dowser (version 2.2.0) CRAN (R 4.3.3), dplyr (version 1.1.4) CRAN (R 4.3.2), DT (version 0.33) CRAN (R 4.3.3), enrichplot (version 1.22.0) Bioconductor, EnsDb.Hsapiens.v79 (version 2.99.0) Bioconductor, ensembldb (version 2.26.1) Bioconductor 3.18, fgsea (version 1.28.0) Bioconductor, forcats (version 1.0.0) CRAN (R 4.2.2), GenomeInfoDb (version 1.38.8) Bioconductor 3.18, GenomicFeatures (version 1.54.4) Bioconductor 3.18, GenomicRanges (version 1.54.1) Bioconductor, ggalluvial (version 0.12.5) CRAN (R 4.2.3), ggplot2 (version 3.5.1) CRAN (R 4.3.3), ggraph (version 2.2.1) CRAN (R 4.3.3), ggtree (version 3.10.1) Bioconductor 3.18, glmGamPoi

(version 1.14.3) Bioconductor 3.18, gridExtra (version 2.3) CRAN (R 4.2.1), gt (version 0.10.1) CRAN (R 4.3.3), gtable (version 0.3.5) CRAN (R 4.3.3), gtools (version 3.9.5) CRAN (R 4.3.2), hdf5r (version 1.3.10) CRAN (R 4.3.3), IRanges (version 2.34.1) Bioconductor, kableExtra (version 1.4.0) CRAN (R 4.3.3), knitr (version 1.46) CRAN (R 4.3.3), lubridate (version 1.9.3) CRAN (R 4.3.1), magrittr (version 2.0.3) CRAN (R 4.2.1), Matrix (version 1.6-5) CRAN (R 4.3.3), Matrix.utils (version 0.9.8) CRAN (R 4.2.1), MatrixGenerics (version 1.14.0) Bioconductor, matrixStats (version 1.3.0) CRAN (R 4.3.3), msigdbr (version 7.5.1) CRAN (R 4.3.3), Nebulosa (version 1.15.0) Bioconductor 3.20, org.Hs.eg.db (version 3.18.0) Bioconductor, org.Mm.eg.db (version 3.18.0) Bioconductor, pacman (version 0.5.1) CRAN (R 4.3.3), paletteer (version 1.6.0) CRAN (R 4.3.3), patchwork (version 1.2.0) CRAN (R 4.3.2), pheatmap (version 1.0.12) CRAN (R 4.3.3), plyr (version 1.8.9) CRAN (R 4.3.1), pryr (version 0.1.6) CRAN (R 4.3.3), purrr (version 1.0.2) CRAN (R 4.3.1), RColorBrewer (version 1.1-3) CRAN (R 4.2.0), Rcpp (version 1.0.12) CRAN (R 4.3.2), ReactomePA (version 1.46.0) Bioconductor, readr (version 2.1.5) CRAN (R 4.3.2), readxl (version 1.4.3) CRAN (R 4.2.3), rhdf5 (version 2.46.1) Bioconductor, rstatix (version 0.7.2) CRAN (R 4.2.2), Rstudioapi (version 0.16.0) CRAN (R 4.3.3), S4Vectors (version 0.38.2) Bioconductor, scales (version 1.3.0) CRAN (R 4.3.2), scoper (version 1.3.0) CRAN (R 4.3.1), Seurat (version 5.1.0) CRAN (R 4.3.3), SeuratData (version 0.2.2) Github (satijalab), SeuratObject (version 5.0.2) CRAN (R 4.3.3), shazam (version 1.2.0) CRAN (R 4.3.1), SingleCellExperiment (version 1.24.0) Bioconductor, sp (version 2.1-4) CRAN (R 4.3.3), stringr (version 1.5.1) CRAN (R 4.3.2), SummarizedExperiment (version 1.32.0) Bioconductor, tibble (version 3.2.1) CRAN (R 4.2.3), tidyr (version 1.3.1) CRAN (R 4.3.3), tidyverse (version 2.0.0) CRAN (R 4.2.2), tigger (version 1.1.0) CRAN (R 4.3.2), viridis (version 0.6.5) CRAN (R 4.3.3), viridisLite (version 0.4.2) CRAN (R 4.2.1), writexl (version 1.5.0) CRAN (R 4.3.3). Additionally the Immcantation workflow for BCR analysis (Version 4.5.0).

For manuscripts utilizing custom algorithms or software that are central to the research but not yet described in published literature, software must be made available to editors and reviewers. We strongly encourage code deposition in a community repository (e.g. GitHub). See the Nature Portfolio guidelines for submitting code & software for further information.

Data

Policy information about availability of data

All manuscripts must include a data availability statement. This statement should provide the following information, where applicable:

- Accession codes, unique identifiers, or web links for publicly available datasets
- A description of any restrictions on data availability
- For clinical datasets or third party data, please ensure that the statement adheres to our policy

Data availability

All data are included in the Supplementary Information or available from the authors, as are unique reagents used in this Article. The raw numbers for charts and graphs are available in the Source Data file. The ScRNA-seq data generated in this study have been deposited in theat GEO database under accession code (GSE275094) and are publicly available here: https://www.ncbi.nlm.nih.gov/geo/query/acc.cgi?acc=GSE275094. Further information and requests for resources contact the corresponding author, Andreia C. Lino (andreia.lino@drfz.de or andreialino79@gmail.comandreialino79@gmail.com).

Research involving human participants, their data, or biological material

Policy information about studies with human participants or human data. See also policy information about sex, gender (identity/presentation), and sexual orientation and race, ethnicity and racism.

Reporting on sex and gender	Sex or gender were not considered in the study design as it was not relevant to our research objectives
Reporting on race, ethnicity, or other socially relevant groupings	We have not collected information on race, ethnicity, or other socially relevant groupings
Population characteristics	Human BM samples were obtained from nine patients undergoing total hip arthroplasty. Human spleens were obtained from five immune thrombocytopenia purpura patients under splenectomy surgery. Human tonsils samples were from six patients under routine tonsillectomy. Human peripheral blood samples were collected from 14 healthy donors. For SLE and control cohorts, peripheral blood samples were collected from 10 patients and eight healthy donors, respectively (Supplementary Table 1). All samples were collected in Charité Universitätsmedizin (Berlin, Germany). The study was approved by the institutional ethics committee of Charité Universitätsmedizin Berlin in accordance with the Declaration of Helsinki. Written informed consent was obtained from all participants prior to sample collection.
Recruitment	Human BM samples were obtained from nine patients undergoing total hip arthroplasty. Human spleens were obtained from five immune thrombocytopenia purpura patients under splenectomy surgery. Human tonsils samples were from six patients under routine tonsillectomy. Human peripheral blood samples were collected from 14 healthy donors. For SLE and control cohorts, peripheral blood samples were collected from 10 patients and eight healthy donors, respectively (Supplementary Table 1).
Ethics oversight	All samples were collected in Charité Universitätsmedizin (Berlin, Germany). Ethics Committee of the Charité Universitätsmedizin Berlin in compliance with the Declaration of Helsinki (EA1/261/09, EA1/009/17, and EA2/010/21).<br>Informed consent was obtained from all donors included in the study.<br>Written informed consent was obtained from all participants prior to sample collection.

Note that full information on the approval of the study protocol must also be provided in the manuscript.

Field-specific reporting

Please select the one below that is the best fit for your research. If you are not sure, read the appropriate sections before making your selection.

- [x] Life sciences
- [ ] Behavioural & social sciences
- [ ] Ecological, evolutionary & environmental sciences

For a reference copy of the document with all sections, see nature.com/documents/nr-reporting-summary-flat.pdf

Life sciences study design

All studies must disclose on these points even when the disclosure is negative.

Sample size	No statistical methods were used to pre-determine sample sizes as our study was not prospective clinical trial. Sample size of human participants were determined by availability of donors in the time of study. Sample size of animal experiments were based on our previous publications and calculated using the G*Power program. We specified the significance level, power of the test and the minimum differences. We know the expected standard deviations of the target variables from comparable experiments. The two-tailed non-parametric Mann–Whitney test was used as the test to be used.
Data exclusions	Poor quality and non-antibody secreting cells were excluded from clustering analysis as in detail describes in the manuscript. The remaining data were not excluded from the analysis.
Replication	All experiments were replicated at least 2-3 times. The replication numbers were described in the corresponding figure legends.
Randomization	Randomization was not applicable as the groups were determined by genotype.
Blinding	Blinding was not applicable due to the nature of the samples analyzed. For human data detailed clinical history was only obtained after analysis. Data were analyzed using predefined pipelines and automated clustering approaches. For flow cytometry data we followed the recommended Guidelines for the use of flow cytometry and cell sorting in immunological studies (PMID: 34910301). For scRNA data we used common packages and workflows.

Reporting for specific materials, systems and methods

We require information from authors about some types of materials, experimental systems and methods used in many studies. Here, indicate whether each material, system or method listed is relevant to your study. If you are not sure if a list item applies to your research, read the appropriate section before selecting a response.

Materials & experimental systems

n/a	Involved in the study
	[x] Antibodies
[x]	Eukaryotic cell lines
[x]	Palaeontology and archaeology
	[x] Animals and other organisms
[x]	Clinical data
[x]	Dual use research of concern
[x]	Plants

Methods

n/a	Involved in the study
[x]	ChIP-seq
	[x] Flow cytometry
[x]	MRI-based neuroimaging

Antibodies

Antibodies used	Mouse antibodies: 1:400 of B220/CD45R-BV711 (clone RA3-6B2, BioLegend, cat. no. 103255); 1:400 of B220/CD45R-PE (clone RA3-6B2, BioLegend, cat. no. 103208); 1:200 of CD2-PE (clone RM2-5, BioLegend, cat. no. 100107); 1:200 of CD2-APC-Vio770 (clone REA959/RM2-5, Miltenyi Biotec, cat. no. 130-115-962); 1:100 of CD3-APC/Cy7 (clone 17A2, BioLegend, cat. no. 100221); 1:200 of CD3-BV421 (clone 145-2C11, BioLegend, cat. no. 100341); 1:100 of CD5-FITC (clone 53-7.3, BD Biosciences, cat. no. 553021); 1:100 of CD5-BV510 (clone 53-7.3, BioLegend, cat. no. 100627); 1:400 of CD11b-Pacific Blue (clone M1/70, BioLegend, cat. no. 101224); 1:200 of CD11b-APC/Cy7 (clone M1/70, BioLegend, cat. no. 101226); 1:100 of CD11c-Pacific Blue (clone N418, BioLegend, cat. no. 117322); 30 µg/ml of CD16/CD32 (Fc block, clone 2.4G2, DRFZ, non-commercial); 1:200 of CD19-BV785 (clone 6D5, BioLegend, cat. no. 115543); 1:200 of CD19-BUV737 (clone 1D3, BD Biosciences, cat. no. 612781); 1:200 of CD20-PE (clone SA275A11, BioLegend, cat. no. 150409); 1:200 of CD21/35-FITC (clone 7G6, BD Biosciences, cat. no. 553818); 1:200 of CD21/35-PE/Cy7 (clone 7E9, BioLegend, cat. no. 123420); 1:400 of CD23-Biotin (clone B3B4, BD Biosciences, cat. no. 553137); 1:400 of CD23-Alexa647 (clone B3B4, BioLegend, cat. no. 101612); 1:200 of CD38-PE (clone 90, BioLegend, cat. no. 102707); 1:400 of CD39-PE/Dazzle594 (clone Duha59, BioLegend, cat. no. 143811); 1:400 of CD39-APC (clone Duha59, BioLegend, cat. no. 143810); 1:200 of CD40-PE (clone 3/23, BioLegend, cat. no. 124609); 1:400 of CD49b-PE (clone HMα2, BioLegend, cat. no. 103506); 1:200 of CD69-PE (clone H1.2F3, BioLegend, cat. no. 104507); 1:100 of CD69-FITC (clone REA937/H1.2F3, Miltenyi Biotec, cat. no. 130-115-574); 1:200 of CD73-PE (clone TY/11.8, BioLegend, cat. no. 127205); 1:100 of CD73-APC-Vio770 (clone REA778/TY/11.8, Miltenyi Biotec, cat. no. 130-111-520); 1:100 of CD80-BV650 (clone 16-10A1, BioLegend, cat. no. 104731); 1:200 of CD80-PE (clone 16-10A1, BioLegend, cat. no. 104707); 1:200 of CD81-PE/Cy7 (clone Eat-2, BioLegend, cat. no. 104913); 1:200 of CD86-BV785 (clone GL-1, BioLegend, cat. no.

105043); 1:200 of CD86-PE (clone PO3, BioLegend, cat. no. 105105); 1:200 of CD117-PE (clone 2B8, BioLegend, cat. no. 105807); 1:100 of CD130-PE (clone 4H1B35, BioLegend, cat. no. 149403); 1:400 of CD138-PE (clone 281-2, BD Biosciences, cat. no. 553714); 1:800 of CD138-BV421 (clone 281-2, BioLegend, cat. no. 142523); 1:200 of CD150-PE (clone TC15-12F12.2, BioLegend, cat. no. 115903); 1:200 of CD326-BV510 (clone G8.8, BioLegend, cat. no. 118231); 1:200 of CD326-APC/Cy7 (clone G8.8, BioLegend, cat. no. 118218); 1:150 of CD352-VioBright B515 (clone REA1097/13G3, Miltenyi Biotec, cat. no. 130-118-731); 1:200 of CD352-PE (clone 330-AJ, BioLegend, cat. no. 134605); 1:100 of CD365-PE (clone RMT1-4, BioLegend, cat. no. 119505); 1:200 of CXCR3-APC (clone CXCR3-173, BioLegend, cat. no. 126512); 1:200 of Igλ-Alexa647 (clone LS136, DRFZ, non-commercial); 1:100 of JAML-Alexa647 (clone 4E10, BioLegend, cat. no. 128506); 1:200 of Ki-67-APC (clone 16A8, BioLegend, cat. no. 652405); 1:150 of LAG-3-PE/Cy7 (clone eBioC9B7W, Thermo Fisher, cat. no. 25-2231-82); 1:400 of Ly6C-PE (clone HK1.4, BioLegend, cat. no. 128007); 1:200 of MHC-II-FITC (clone M5/114, DRFZ, non-commercial); 1:200 of TIGIT-PE (clone 1G9, BioLegend, cat. no. 142103); LEGENDScreen Mouse PE Kit [BioLegend, cat. no. 700005]; 1:400 of VH11-PE (clone P18-3H7, gift from Kyoko Hayakawa, non-commercial) (PMIDs: 17889506, 30005826); 1:100 of Vk14-Pacific Orange (clone P18-13B5, gift from Kyoko Hayakawa, non-commercial) (PMIDs: 17889506, 30005826); 1:200 of VH12-APC (clone 5C5, gift from Klaus Rajewsky, non-commercial) (PMIDs: 30765568, 30005826).

DNA-barcoded antibodies: 1:400 of CD19-TotalSeq-C0093 (clone 6D5, BioLegend, cat. no. 115571); 1:100 of CD39-TotalSeq-C0834 (clone Duha59, BioLegend, cat. no. 143815); 1:400 of CD40-TotalSeq-C0903 (clone 3/23, BioLegend, cat. no. 124635); 1:200 of CD45R/B220-TotalSeq-C0103 (clone RA3-6B2, BioLegend, cat. no. 103273); 1:200 of CD69-TotalSeq-C0197 (clone H1.2F3, BioLegend, cat. no. 104551); 1:200 of CD73-TotalSeq-C0077 (clone TY/11.8, BioLegend, cat. no. 127237); 1:200 of CD80-TotalSeq-C0849 (clone 16-10A1, BioLegend, cat. no. 104755); 1:200 of CD183/CXCR3-TotalSeq-C0228 (clone CXCR3-173, BioLegend, cat. no. 126545); 1:200 of CD223/LAG-3-TotalSeq-C0378 (clone C9B7W, BioLegend, cat. no. 125237); 1:200 of CD326-TotalSeq-C0449 (clone G8.8, BioLegend, cat. no. 118243); 1:400 of CD117/c-Kit-TotalSeq-C0012 (clone 2B8, BioLegend, cat. no. 105851); 1:400 of CD150/SLAM-TotalSeq-C0203 (clone TC15-12F12.2, BioLegend, cat. no. 115947); 1:400 of Ly6C-TotalSeq-C0013 (clone HK1.4, BioLegend, cat. no. 128051).

Human monoclonal antibodies: 1:50 of CD3-BUV395 (clone UCHT1, BD Biosciences, cat. no. 563546); 1:50 of CD14-BUV395 (clone M5E2, BD Biosciences, cat. no. 740286); 1:20 of CD19-BV711 (clone SJ25C1, BD Biosciences, cat. no. 563038); 1:20 of CD20-BV510 (clone 2H7, BioLegend, cat. no. 302340); 1:25 of CD27-BV786 (clone L128, BD Biosciences, cat. no. 563327); 1:500 of CD38-APC/Cy7 (clone HIT2, BioLegend, cat. no. 303534); 1:20 of CD39-BV421 (clone A1, BioLegend, cat. no. 328214); 1:20 of CD81-FITC (clone 5A6, BioLegend, cat. no. 349504); 1:20 of CD130-PE/Cy7 (clone 2E1B02, BioLegend, cat. no. 362007); 1:20 of CD138-BUV737 (clone MI15, BD Biosciences, cat. no. 564393); 1:20 of CD326-BV605 (clone 9C4, BioLegend, cat. no. 324224); 1:20 of FcR Blocking Reagent (Miltenyi Biotec, cat. no. 130-059-901).

Additional antibodies: 1:1000 of Goat Anti-Mouse Ig(H+L) (polyclonal, SouthernBiotech, cat. no. 1010-01); 1:1000 of Anti-Mouse IgM-AP (polyclonal, SouthernBiotech, cat. no. 1020-04); 1:1000 of Anti-Mouse IgA-AP (polyclonal, SouthernBiotech, cat. no. 1040-04); 1:1000 of Anti-Mouse IgG-AP (polyclonal, SouthernBiotech, cat. no. 1030-04); 1:10 of Anti-PE MicroBeads (Miltenyi Biotec, cat. no. 130-048-801); 1:10 of Anti-PE MicroBeads UltraPure (Miltenyi Biotec, cat. no. 130-105-639); 1:400 of Streptavidin-BV650 (BioLegend, cat. no. 405232); 1:400 of Streptavidin-Alexa488 (Thermo Fisher, cat. no. S32354).

Validation

Most antibodies in this study were commercial antibodies. These antibodies were validated by their manufacturers (find technical data sheets by the catalog numbers) and further in-house testing. Other antibodies, including VH11 (Clone P18-3H7) and Vk14 (Clone P18-13B5) antibodies were the gift from Prof. Kyoko Hayakawa (Fox Chase Cancer Center, Philadelphia, USA) (PMIDs: 17889506, 30005826);. VH12 (Clone 5C5) antibody was the gift from Prof. Klaus Rajewsky (MDC Berlin) (PMIDs: 30765568, 30005826). These antibodies were validated by previous reports and also in-house testing as described to the Guidelines for the use of flow cytometry and cell sorting in immunological studies (PMID: 34910301).

Animals and other research organisms

Policy information about studies involving animals; ARRIVE guidelines recommended for reporting animal research, and Sex and Gender in Research

Laboratory animals

C57BL/6 (DRFZ Berlin, Germany), Cd21-cre-ROSA26-STOP-eYFP (generated by crossing Cd21-cre (Strain #:006368) with ROSA26-STOP-eYFP (Jackson, Strain #:00614), Cγ1-cre-ROSA26-STOP-eYFP (generated by crossing Cγ1-cre (from K. Rajewsky, MDC Berlin, Germany) (PMID: 16651521) with ROSA26-STOP-eYFP (Jackson, Strain #:00614), prdm1eGFP (from S. Nutt, Walter and Eliza Hall Institute, Melbourne, Australia) (PMID: 15492122), Rag-/- (Jackson, Strain #:008449), Tcrβδ-/- (Jackson, Strain #:002122), and Sle123 (Jackson, Strain #:007228) (all C57BL/6 background) mice were bred under specific pathogen-free conditions at the DRFZ, Charité Universitätsmedizin (Berlin, Germany) and Institut Necker-Enfants Malades (Paris, France). Germ-free (GF) and specific-pathogen free (SPF) C57BL/6 mice were also bred at the German Institute of Human Nutrition, Potsdam, Germany. Experimental and control animals were housed and bred in the same animal facility. Environmental conditions were standardized, with a 12-hour light/12-hour dark cycle controlled by automated timers with an adjustable twilight phase. The temperature was maintained at 22 ± 2°C, and relative humidity was kept between 45–65%. Sle123 mice were examined for proteinuria twice per week using Multistix (Siemens) from week 24 onwards. Sick Sle123 mice (26-30 weeks old) were euthanized for analysis when proteinuria >100 mg/dL. Young Sle123 mice (6-10 weeks old) without proteinuria were included as healthy control mice. Other mice were between 9-20 weeks old, unless otherwise stated. Both male and female mice were used in all experiments, with gender-matched controls included, except in experiments involving Sle123 mice, where only females were used since most males do not develop the lupus phenotype. This approach reflects the higher prevalence of SLE in women compared to man. All animal experiments were reviewed and approved by Landesamt für Gesundheit und Soziales Berlin (LAGeSo, Berlin) under licenses T0344/17, G0072/20, and G0159/23. Mice were euthanized by cervical dislocation.

Wild animals

This study didn't use any wild animals

Reporting on sex

Both male and female mice were used in all experiments, with gender-matched controls included, except in experiments involving Sle123 mice, where only females were used since most males do not develop the lupus phenotype. This is consistent with human because SLE affects mainly women at a childbearing age, with a ratio of 9:1 (women: man).

Field-collected samples	This study didn't involve sample collection from the field.
Ethics oversight	All animal experiments were reviewed and approved by Landesamt für Gesundheit und Soziales Berlin (LAGeSo, Berlin) under licenses T0344/17, G0072/20, and G0159/23.

Note that full information on the approval of the study protocol must also be provided in the manuscript.

Plants

Seed stocks	N/A
Novel plant genotypes	N/A
Authentication	N/A

Flow Cytometry

Plots

Confirm that:

- [x] The axis labels state the marker and fluorochrome used (e.g. CD4-FITC).
- [x] The axis scales are clearly visible. Include numbers along axes only for bottom left plot of group (a 'group' is an analysis of identical markers).
- [x] All plots are contour plots with outliers or pseudocolor plots.
- [x] A numerical value for number of cells or percentage (with statistics) is provided.

Methodology

Sample preparation	Mouse and human sample preparation were described in detail in Method section
Instrument	Flow cytometry (FACS): BD FACSymphony and BD LSRFortessa flow cytometer<br>Cell sorter: BD FACS sorter Aria II
Software	BD FACS Diva Software V8.0 and Flowjo version10 (BD)
Cell population abundance	We collected data on at least 5,000,000 cells per experimental condition.<br>The purity of sorted cell populations were around 95% , determined by flow cytometric analysis of post-sort samples.
Gating strategy	For human samples:<br>Cells were first gated by FSC/SSC to exclude debris, followed by gating FSC-A and FSC-H to eliminate non singlets, subsequently, dead cells and CD3+CD14+ cells were excluded. Then, target cells (CD27+CD38+) were gated for further analysis of CD19+ and CD19- cell populations.<br><br>For mouse samples:<br>For flow analysis: Cells were first gated by FSC/SSC to exclude debris, followed by gating FSC-A and FSC-H to eliminate non singlets, subsequently, dead cells were excluded. Then, plasma cells (CD39+CD138+ or CD39+CD130+ or CD39+Blimp1+ or CD138hi) were gated for further analysis of CD19+ and CD19- cell populations as well as antigen-specific plasma cell subsets within CD19+ and CD19- plasma cell populations<br>For plasma cell sorting: Cells were first gated by FSC/SSC to exclude debris, followed by gating FSC-A and FSC-H to eliminate non singlets, subsequently, dead cells and CD11b+CD11c+CD3+were excluded. Then, total plasma cells (CD138+CD81+) were gated and sorted for single-cell RNA sequencing, CD19+ plasma cells (CD138+CD39+CD19+) and CD19- plasma cells (CD138 +CD39+CD19-) were sorted for ELISPOT, ELISA and adoptive transfer experiments.

- [x] Tick this box to confirm that a figure exemplifying the gating strategy is provided in the Supplementary Information.
